# Developing Physical Assessment Skills in Pharmacy Students through Participation in a Creative Movement Workshop: An Interdisciplinary Study between Pharmacy and Dance

**DOI:** 10.3390/pharmacy8030142

**Published:** 2020-08-11

**Authors:** Amber Wesner, Ting-Yu Chen

**Affiliations:** 1 Bernard J Dunn School of Pharmacy, Shenandoah University, 1775 North Sector Court, Winchester, VA 22601, USA; 2 Shenandoah Conservatory, Shenandoah University, 1460 University Drive, Winchester, VA 22601, USA; tchen@su.edu

**Keywords:** physical assessment, pharmacy education, creative movement, interdisciplinary

## Abstract

The role a pharmacist plays in the care of patients is continually changing and expanding. Most recently, there is movement towards including pharmacists in the physical assessment of patients. We developed a creative movement workshop with the purpose of increasing students’ levels of comfort with touch, ability to interpret non-verbal mannerisms, to increase empathy for the patient, and to increase student comfort in conducting physical exams. In this interventional study, surveys were administered to third year pharmacy students, before and after the creative movement workshop, in order to assess participant’s change in level of comfort with a variety of behaviors needed to conduct effective physical assessment. The two hour workshop involved: partner stretching, mirroring, and creative spatial exploration between bodies. The 11-item survey evaluated students’ perceptions on touch, nonverbal communication, and sharing personal space. Our results showed that the level of comfort improved for the ability to give touch (*p* = 0.001), the ability to receive touch (*p* = 0.002), and the ability to share personal space (*p* = 0.001). Participants commented that the workshop increased their understanding of how important confidence is when performing physical assessment and reported an increased appreciation for how much non-verbal mannerisms can communicate to another. This study explores how an interdisciplinary workshop between pharmacy and dance has the potential to increase student effectiveness as future healthcare professionals, by targeting skills not often focused on within traditional pharmacy curriculums, including: sharing personal space, displaying empathy, and providing a comforting and confident touch.

## 1. Introduction

Pharmacists are being called on increasingly to participate in the physical assessment of patients [1,2,3]. As a result, curriculums must evolve, and activities need to be implemented, in order to enhance students’ levels of comfort with providing these types of interventions [4,5,6,7,8]. Failure to develop effective physical assessment skills in pharmacy students will result in students struggling during their experiential rotations, limit the role of the pharmacist on interprofessional teams, and potentially harm patient relationships [9,10]. In the article by Chua, et al., “Pharmacist performance of physical assessment: perspectives of clinical pharmacists working in different practice settings,” they include opinions from current pharmacists, where it is stated that the non-verbals displayed by a pharmacist influence if a patient believes a pharmacist is competent at performing physical assessments [11]. The article also offers support for providing formalized training and practice for pharmacy students and residents in this area [9,12,13]. In another study that investigated pharmacy students’ perceptions of a physical assessment course and how it was taught, 46% of respondents disagreed or were neutral that the topics covered were relevant to pharmacy practice [14]. These findings highlight the need to educate students on the pharmacist’s role in physical assessment and highlights the need to improve skills such as non-verbal communication and empathy, which rarely are addressed within pharmacy curriculums [11,14,15,16,17].

In order to provide effective physical assessment, clinicians must be calm, confident, empathetic to patient needs, able to read body language, and able to share personal space in a respectful and comfortable manner [4,11,18]. In an effort to develop these skills, we organized a movement workshop in order to get students outside of their comfort zone and to target enhancing their ability to empathize. Our institution has a vast array of programs, including dance and pharmacy. This workshop was inspired by all of the cross-disciplinary work that occurs between programs in an effort to target the extensive variety of skills students need to be successful. It was recognized that including a dance-inspired creative movement workshop within the pharmacy program held the potential to increase self-awareness and potentially develop physical assessment skills in pharmacy students. This was based on the observation that students appeared quite uncomfortable interacting with patients physically while in lab and hearing student comments where they felt uncomfortable conducting physical assessment. This study highlights a unique approach to target and potentially develop self-awareness and physical assessment skills in students.

The primary objective of this study was to determine if pharmacy students will become more comfortable at providing physical touch, sharing personal space, and interpreting nonverbal communication after completing a workshop focusing on physical contact and spatial awareness. The purpose of the workshop was to provide experiential learning for pharmacy students on the topics of self-awareness, observation skills for effective nonverbal communication, and responsiveness to the needs of the self and others [19,20]. Thus, creating a more effective approach to patient evaluation for the prospective health care provider. Ultimately, the goal was for pharmacy students to translate the increased self-awareness gained from the workshop to become more comfortable performing physical assessment on patients.

## 2. Materials and Methods

### 2.1. Design

This was a prospective interventional study evaluating the effects of a creative movement workshop on the self-reported level of comfort with providing touch and physical assessment among pharmacy students.

### 2.2. Sample

**Figure 1:**
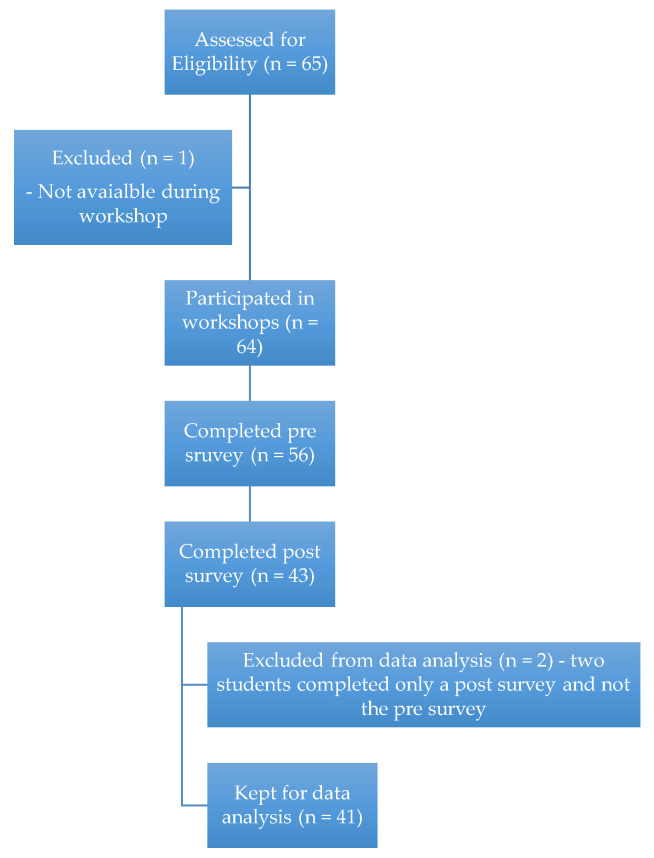
Flowchart of participants.

Students could participate if they were a third year Doctor of pharmacy student, completed the study orientation, and provided informed consent. The number of students screened for eligibility, number who completed the pre and post surveys, and the number kept for analysis can be followed in Figure 1.

### 2.3. Intervention

Participants were first introduced to the study at an orientation meeting. The study orientation consisted of a group meeting where an overview of the study was provided, and students were given the opportunity to ask questions and to review the informed consent document. Students were asked to return the form within 48 h of the group meeting if they chose to participate in the study. After deciding to participate and signing the informed consent, students received a study ID number and link to the electronic pre survey (survey questions can be viewed in Table 1). 

After completion of the pre survey, students then participated in one of two creative movement workshops. One workshop was facilitated by Ting-Yu Chen, a dance professor and Associate Dean of Student Affairs for the Shenandoah Conservatory, and the other was facilitated by the Stuart Pimsler Dance Theater, a dance company in residence at Shenandoah University during the study period. The Stuart Pimsler Dance Theater’s philosophy and mission includes using the arts to enhance healthcare. Both workshops had the same purpose: to use non-verbal creative movement exercises and expression to enhance the provider-patient relationship. Workshops were held in January and February of 2018.

During the 120-min workshop, students engaged in mindful meditation, gentle stretching, as well as creative movement exercises used to enhance self-awareness through personal expression and interpretation. Activities included mirroring, stretching, both alone and with partners, and manipulating personal space, which increased in difficulty by progressively adding to the size of the groups participating. Negative space was the technique utilized to explore personal space. This required a participant to create a structure with their body, thus forming positive space, and a second participant to fill the negative space around the first individual. Participants in negative space exercises may find themselves in unusual positions within each other’s personal space as they create shapes around one another. Thus, students have a silent dialogue with each other, only involving their bodies. Not only can negative space exercises involve two individuals, but they can also involve larger groups, resembling an interprofessional team dynamic. Discussion of the exercises occurred frequently throughout the sessions, and students were asked to identify how the exercises could be related to pharmacy practice and patient care. The series of exercises were completed in respect to each student’s individual range of motion without any forceful manipulation, and no prior movement experience was needed. The workshop focused on developing a keen sense of physical observation, enhancing movement range, flexibility, observation skills, and an awareness of the body in space. These activities were designed to facilitate tactile sensitivity and movement coordination necessary for appropriate patient evaluation, while increasing empathy to what a patient experiences during a physical evaluation. The workshop was meant to prepare the clinician’s body to be energized, present, and ready to engage patients and healthcare teammates with ease and confidence. In addition, these skills were targeted to assist the clinician in interpreting nonverbal cues from their patients and interprofessional healthcare team members.

Upon completion of the workshop, participants were asked to complete an electronic post survey. Students were also asked to fill out a Workshop Evaluation to gauge their opinion on the effectiveness of the body awareness workshop, the success and weaknesses of their workshop experience, and their suggestions for future improvement.

### 2.4. Survey Tool

The survey tool used was created by the researchers. It had been tested for validity and reliability with a test group that used the same survey for a previous movement seminar not reported here. It was administered through Survey Monkey^®^ and took less than 5 min to complete. The scale included with the survey was a 5-point Likert scale. Scores of one were interpreted as strongly disagreeing with the given statement, scores of three were interpreted as neutral and without strong feeling either way, and scores of five were interpreted as strongly agreeing with the given statement.

### 2.5. Data Analysis

Data from the survey was downloaded into an Excel spreadsheet in order to enter it effectively into statistical software. Data were analyzed using SPSS (SPSS statistics, version 25, IBM Corp., Armonk, NY, USA). Pre and post survey data, which were paired ordinal data, were evaluated using the Wilcoxon Signed Rank test. A value less than 0.05 was considered significant.

### 2.6. Ethics

This study was approved by Shenandoah University’s Institutional Review Board.

## 3. Results

The identified pharmacy class had sixty-five students total, with sixty-four available to participate in the workshop. Of these, thirty-five students were located on the Fairfax, VA campus, and twenty-nine were located on the Winchester, VA campus. Fifty-six students completed the pre survey and forty-three students completed the post survey, representing an 88% and 67% participation rate on the two surveys, respectively. Only participants who completed both the pre and post surveys were kept for data analysis, which resulted in a sample size of forty-one paired survey results. Twenty-five surveys represent the Fairfax campus and sixteen surveys represent the Winchester campus.

Pooled results of the pre and post surveys can be viewed in Table 1. Significant improvements were noted for three questions. Students noted having less stress related to giving touch (*p* = 0.001), less stress receiving touch (*p =* 0.002), and were more comfortable sharing personal space (*p =* 0.001). Results also showed improved scores for students feeling in touch with their body, being at ease in their own skin, and ability to read nonverbal reactions. However, these scores did not show a statistically significant difference.

Subgroup data are included in Table 2 and Table 3, looking at each campus separately. Survey results for only the Fairfax campus can be viewed in Table 2, and results for the Winchester campus only can be viewed in Table 3. A statistical analysis was not conducted for each campus separately, data were provided for informational purposes only. As in the overall analysis, questions which showed the greatest change were the level of comfort in sharing personal space and the level of anxiety decreasing with giving and receiving of touch. These changes were more notable for the Fairfax campus.

Demographic data can be viewed in Table 4. The average age of participants was 26 ± 3 years, and 76% were female. The majority of students, 59%, self-identified as Caucasian, followed by 24% identifying as Asian. Self-reported differences in race were noted between campuses, with 94% of Winchester students identifying as Caucasian, and only 36% of Fairfax students identifying as Caucasian. The largest race demographic in Fairfax was Asian, representing 40% of participants.

Evaluation data regarding the organization and content of the workshop can be viewed in Table 5. Scores ranged from 3.9 to 4.4, indicating that students agreed with the questions, thought the workshop was well organized and was explained well, the ideas were conveyed effectively, that they gained an increased understanding of the content, and that they will be able to apply the skills learned as a future pharmacist.

## 4. Discussion

Physical assessment is an emerging skill for pharmacists that is now a requirement in pharmacy education, as it is encompassed within the Pharmacists’ Patient Care Process and is part of the proposed Entrustable Professional Activities for pharmacy practice [1,21,22,23]. Even with physical assessment education being provided, more work is needed to: (1) educate other healthcare professionals on how pharmacists can contribute when it comes to physical assessment, (2) improve pharmacists’ comfort in participating in this type of activity, and (3) establishing trust with patients so that pharmacists can provide them with this service. Several studies have shown evidence that pharmacists are uncomfortable with performing physical assessment and that other healthcare providers do not necessarily support pharmacist’s participation in this activity.

In a study by Barry et al., which evaluated pharmacist’s perceptions of providing physical exams, they found that the three most common barriers to providing physical assessment identified by pharmacists were: feeling uncomfortable performing physical exams, lack of training, and a perceived impression that patients would be uncomfortable with a pharmacist completing this type of intervention [24]. In a second study, by Breault, et al., which assessed licensed pharmacist’s impressions of physical assessment skills in an institutional setting before and after a workshop, the most common barriers identified as to why physical assessment is not performed in practice were: lack of comfort, lack of training, and deeming it unnecessary since other professionals complete this skill [24]. Additionally, when pharmacists scored their level of comfort before the workshop, the average score was a one, which translated to “not confident” in performing physical assessments [25]. Finally, in a study by Chevalier et al., where physicians and nurses were surveyed on the services that clinical pharmacists provide, none of the physicians surveyed indicated that having pharmacists complete physical assessment would be beneficial to their practice [26]. Each of these studies highlights why the content of our study is necessary. It is essential that we find ways to increase the level or comfort and confidence for pharmacists in completing physical assessment. Our study aimed to address the barrier of lack of comfort as a reason why physical assessment is not completed regularly by pharmacists.

Our study showed that participation in a creative movement workshop could improve the level of comfort a student feels when providing touch, receiving touch, and sharing their personal space, all of which are needed to be an effective provider of physical exams. In addition to objectively scoring their level of comfort in the survey, students were also able to add comments about the workshop and what they learned from this experience. Several themes in the responses emerged, including an increased appreciation for how much non-verbals can communicate, an increased understanding of what a patient experiences during a physical assessment, recognizing the importance of displaying confidence to help others establish trust and comfort, and a recognition that the anxiety level of the person providing the exam will affect the anxiety of the person receiving the exam.

From our interpretation of the student comments, we noted an increase in empathy and an increase in understanding of what it is like to receive touch. Most, if not all of these students, are young and healthy, and have not had to experience a patient assessment apart from a standard physical for school requirements. Based upon these comments and the study results, we believe that students benefited from the workshop and that they will be able to better relate to their patients. The workshop required students to share their personal space as well as to participate in giving and receiving touch, thus resembling the intimacy of a clinical exam between a healthcare provider and a patient. The activities allowed students to see both the provider and patient perspective of touch in a low stress and low stakes environment.

Our workshop and study principle of using creative movement exercises to improve physical assessment skills, and thus improve the provider-patient relationship, is unique to pharmacy-related literature, but the concept is supported by previous research. Several studies highlight the importance of the quality of touch in the practitioner-patient relationship, and how much of a patient’s perception of a healthcare provider is based off of the touch they receive [27,28]. In the study by Consedine et al., they found that care, professionalism, confidence, competence, trust, and engagement were all communicated through the touch that an osteopathic practitioner provided to their patients [27]. Thus, since so many skills can potentially be communicated through touch, utilizing creative movement to improve pharmacy student’s confidence in providing touch and empathy for the patient experience is an exercise well worth our time, and is something that can pay dividends as the student proceeds into practice. In addition, literature suggests that the act of mirroring may enhance empathy [29]. Given the findings and comments from our participants of an increased awareness and understanding of the patients’ perspective, our study supports this claim as mirroring was a key component of the workshop. Overall, using creative movement/dance exercises to directly enhance physical assessment skills is a novel concept. However, the self-awareness and types of skills (giving/receiving touch, sharing personal space, and reading nonverbal cues) that creative movement can address can improve the skills we are trying to target. The few studies found do show that touch and empathy contribute to a positive practitioner-patient relationship, and our study investigated a new approach to developing these skills within pharmacy students [30].

This study is limited by several factors. First, our sample size was small and this was conducted at only one University, thus limiting its generalizability. Additionally, while showing improved comfort levels in providing physical assessment skills is important, it would also be helpful to objectively evaluate if participation in the workshop actually improved student’s physical assessment skills when faced with a patient. While students will complete physical evaluation of patients later in the curriculum, no direct observation of these skills before or after the workshop was done.

In the future, it would be recommended to perform a similar study, but to gather patient feedback on change in student skill and behavior before and after participating in the workshop. Additionally, a similar study could be designed where one study group participates in a creative movement workshop and one proceeds through the traditional curriculum to investigate how much the workshop influences physical assessment skills.

## 5. Conclusions

A creative movement workshop improved pharmacy student’s self-reported level of comfort with providing touch, receiving touch, and with sharing their personal space with others. Open-ended responses provided by participants indicated that they developed an increased level of understanding for what a patient goes through during a physical exam. As pharmacists’ roles on interdisciplinary teams continue to expand, any activities that develop pharmacists’ physical assessment skills and confidence should be explored. Results of this study show that a creative movement workshop can increase the level of comfort in the giving and receiving of touch. Overall, there is limited literature investigating using creative movement to increase empathy, confidence, and comfort in providing physical assessment and this study outlines a novel approach to addressing these skills.

## Figures and Tables

**Table 1:** Survey Results of Entire Study Group.

Students Were Asked to Rate Their Answers on a Likert Scale Where 1 = Strongly Disagree; 2 = Disagree; 3 = Neutral; 4 = Agree; 5 = Strongly Agree					
Question	Pre-Survey (*n* = 41)		Post-Survey (*n* = 41)		*p*-Value
	Mean	Median	Mean	Median	
I am in touch with my feelings and body sensations.	3.8	4	3.9	4	0.450
I have anxiety giving touch to others with whom I am not closely familiar.	3.6	4	3.0	3	0.001
I have anxiety with receiving touch from others with whom I am not closely familiar.	3.7	4	3.0	3	0.002
I am comfortable sharing my personal space with others whom I am not closely familiar.	2.6	2	3.3	4	0.001
I am a good observer of others’ emotions and reactions.	4.2	4	4.2	4	1.000
I am good at listening to others in a conversation.	4.2	4	4.3	4	0.071
I am not good at reading the nonverbal behaviors of others.	2.0	2	1.9	2	0.302
I am comfortable maintaining eye contact in a conversation with someone that I am not closely familiar with.	3.9	4	3.9	4	0.864
When working as a team member, I am comfortable being a leader.	3.7	4	3.9	4	0.142
When working as a team member, I am comfortable being a follower.	3.7	4	3.9	4	0.083
I feel at ease in my own skin.	3.8	4	4.0	4	0.151

**Table 2:** Results of Survey for Fairfax campus participants only.

Students Were Asked to Rate Their Answers on a Likert Scale Where 1 = Strongly Disagree; 2 = Disagree; 3 = Neutral; 4 = Agree; 5 = Strongly Agree				
Question	Pre-Survey (*n* = 25)		Post-Survey (*n* = 25)	
	Mean	Median	Mean	Median
I am in touch with my feelings and body sensations.	3.7	4	4.0	4
I have anxiety giving touch to others with whom I am not closely familiar.	3.4	4	2.6	2
I have anxiety with receiving touch from others with whom I am not closely familiar.	3.5	4	2.6	2
I am comfortable sharing my personal space with others whom I am not closely familiar.	2.6	2	3.6	4
I am a good observer of others’ emotions and reactions.	4.1	4	4.2	4
I am good at listening to others in a conversation.	4.0	4	4.2	4
I am not good at reading the nonverbal behaviors of others.	1.9	2	1.9	2
I am comfortable maintaining eye contact in a conversation with someone that I am not closely familiar with.	3.9	4	3.9	4
When working as a team member, I am comfortable being a leader.	3.6	4	3.8	4
When working as a team member, I am comfortable being a follower.	3.7	4	3.8	4
I feel at ease in my own skin.	4.2	4	4.2	4

**Table 3:** Results of Survey for Winchester campus participants only.

Students Were Asked to Rate Their Answers on a Likert Scale Where 1 = Strongly Disagree; 2 = Disagree; 3 = Neutral; 4 = Agree; 5 = Strongly Agree				
Question	Pre-Survey (*n* = 16)		Post-Survey (*n* = 16)	
	Mean	Median	Mean	Median
I am in touch with my feelings and body sensations.	4.0	4	3.7	4
I have anxiety giving touch to others with whom I am not closely familiar.	3.9	4	3.6	4
I have anxiety with receiving touch from others with whom I am not closely familiar.	4.0	4	3.7	4
I am comfortable sharing my personal space with others whom I am not closely familiar.	2.7	2	2.9	3
I am a good observer of others’ emotions and reactions.	4.2	4	4.2	4
I am good at listening to others in a conversation.	4.4	4	4.5	4.5
I am not good at reading the nonverbal behaviors of others.	2.1	2	1.8	2
I am comfortable maintaining eye contact in a conversation with someone that I am not closely familiar with.	3.9	4	3.9	4
When working as a team member, I am comfortable being a leader.	3.8	4	4.1	4
When working as a team member, I am comfortable being a follower.	3.6	4	3.9	4
I feel at ease in my own skin.	3.3	3.5	3.7	4

**Table 4:** Demographic Information.

Characteristic	Total	Winchester Campus	Fairfax Campus
	(*n* = 41)	(*n* = 16)	(*n* = 25)
Age (yrs)	26 ± 3	25 ± 2	27 ± 3
Race (n)			
Caucasian	24	15	9
Black	3	0	3
Asian	10	0	10
Persian	1	0	1
Multi-Racial	3	1	2
Gender (% Females)	76%	62.50%	84%
Have you received body work? i.e., massage, Reiki, meditation, Yoga, T’ai Chi, Qigong, Pilates, etc. (n)			
Not at all	19	8	11
1–5 times	13	4	9
6–10 times	1	0	1
>10 times	8	4	4
Do you have prior experience as a provider for body work or energy work? i.e., massage, Reiki, meditation, Yoga, T’ai Chi, Qigong, Pilates, etc. (n)			
Yes	1	1	0
No	40	15	25
Do you have prior experience performing physical assessments?			
Yes	27	13	14
No	14	3	11

**Table 5:** Workshop Evaluation.

Students Were Asked to Rate Their Answers on a Likert Scale Where 1 = Strongly Disagree; 2 = Disagree; 3 = Neutral; 4 = Agree; 5 = Strongly Agree			
Question	Total Group	Fairfax Only	Winchester Only
The workshop was well organized.	4.4	4.5	4.3
The facilitator helped me to understand how the workshop material related to a pharmacist’s approach to patient management and evaluation.	4.3	4.6	3.9
The facilitator conveyed ideas effectively and clearly.	4.4	4.6	4.1
The workshop material was informative.	4.2	4.4	3.7
The workshop material was easy to understand.	4.4	4.5	4.1
My understanding of the subject matter has improved as a result of participating in the workshop.	4.1	4.4	3.7
I gained usable skills from the workshop.	3.9	4.3	3.3
I will be able to apply the skills I have learned in the workshop as a future pharmacist.	4.0	4.4	3.4
